# Intravitreal Fluocinolone Acetonide (ILUVIEN) Implant for the Treatment of Refractory Cystoid Macular Oedema After Retinal Detachment Repair

**DOI:** 10.4274/tjo.34966

**Published:** 2018-06-28

**Authors:** Fadi Alfaqawi, Ambreen Sarmad, Kholoud Ayesh, Arijit Mitra, Ash Sharma

**Affiliations:** 1 City Hospital, Birmingham and Midland Eye Centre, Ophthalmology Clinic, Birmingham, United Kingdom; 2 Alquds University Faculty of Medicine, Department of Ophthalmology, Abu Dis, Palestine

**Keywords:** Cystoid macular oedema, retinal detachment repair, ILUVIEN

## Abstract

Cystoid macular oedema (CMO) is one of the most frequent postoperative macular complications to cause partial visual recovery after successful retinal detachment (RD) repair. Refractory CMO is difficult to treat and many strategies have been employed with varying degrees of success. We report for the first time the use of ILUVIEN implant to treat refractory CMO after successful RD repair. A 65-year-old female presented with right eye full-thickness macular hole and underwent pars plana vitrectomy, internal limiting membrane peeling and cryotherapy with gas tamponade with 12% C3F8. She subsequently developed right eye macula-on RD and proliferative vitreoretinopathy and required multiple procedures for successful retinal reattachment. Later, she developed CMO that responded to intravitreal triamcinolone injections and intravitreal dexamethasone 0.7-mg implants but recurrence of CMO continued to be a problem. After receiving ILUVIEN intravitreal implant, her visual acuity improved and CMO resolved without recurrence for 13 months. Refractory CMO after RD repair is difficult to treat and in a quarter of cases will not improve without treatment. Our case shows that a single ILUVIEN implant maintained anatomical dry fovea and improved vision. This also demonstrates that ILUVIEN is an effective management strategy to reduce the need for repeated treatments.

## Introduction

There are several pre- and postoperative factors that may influence visual outcome after successful retinal detachment (RD) repair. The most important preoperative factors are visual acuity (VA) and the duration of the RD. Cystoid macular oedema (CMO) and epiretinal membranes are the main postoperative factors and CMO appears to be the most frequent postoperative macular complication to cause partial visual recovery after successful RD repair.^1^

The exact aetiology of CMO after RD repair is unclear but inflammation is thought to be an important mechanism.^2,3^ Spontaneous resolution of CMO within 2 years postoperatively has been reported in up to 76% of cases.^4^ Many strategies have been employed to manage CMO after RD surgery, with varying degrees of success. Different anti-inflammatory medications have been used, including non-steroidal anti-inflammatory medications and topical, periocular and intravitreal corticosteroids.^3,5,6^

ILUVIEN implant (non-biodegradable 0.2 µg/d fluocinolone acetonide; Alimera Sciences, Inc.) is a sustained-release intravitreal steroid lasting up to 36 months that has been approved in the UK to treat chronic refractory CMO in pseudophakic eyes unresponsive to available therapies.^7^

We report for the first time the use of ILUVIEN implant to treat highly refractory CMO after successful RD repair and the outcomes of 20-month follow-up period after ILUVIEN implant.

## Case Report

A 65-year-old female presented to our tertiary eye centre with 6 weeks’ history of painless right eye vision distortion and no history of eye injury or trauma. On examination, VA was 6/24 in the right eye and 6/5 in the left eye. Following slit-lamp biomicroscopy and fundoscopy a diagnosis of right eye full-thickness macular hole was made and optical coherence tomography showed right eye cuff of subretinal fluid, left eye epiretinal membrane and posterior vitreous detachment. Ten years before presentation she had uneventful bilateral phacoemulsification and intraocular lens implantation with no other significant past ocular or medical history.

Eight weeks later, she underwent right eye pars plana vitrectomy, internal limiting membrane peeling and cryotherapy with C3F8 12% gas tamponade. Two weeks postoperatively, she exhibited a flat retina, closed macular hole and her VA had improved to 6/18 with normal intraocular pressure (IOP). Unfortunately, 7 weeks postoperatively, she developed right eye macula-on RD due to proliferative vitreoretinopathy (PVR) in the inferior retina. RD repair was done within 3 days with silicone oil (Densiron 68) tamponade and retinectomy to release the PVR. After 4 months, VA of the right eye after removal of silicone oil was 6/12 with flat retina and closed macular hole. 

**Figure 1:**
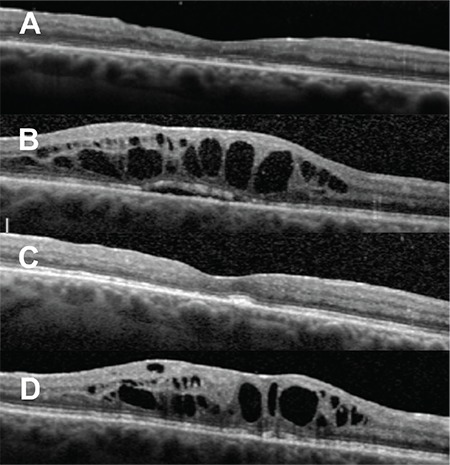
Optical coherence tomography images. A) One month after the first intravitreal triamcinolone injection. B) One month before the first Ozurdex implant, C, D) One month and 5 months after the first Ozurdex implant, respectively

Four months later, her VA declined to 6/36 in the right eye and remained 6/5 in the left eye and fundus fluorescein angiogram confirmed severe right eye CMO. She underwent right eye posterior sub-Tenon’s triamcinolone injection and was started on ketorolac trometamol eye drops (Acular) 3 times/day and oral acetazolamide 250 mg slow-release 2 times/day. Treatment of the CMO during the follow-up period is summarised in Table 1. She received 3 posterior sub-Tenon’s triamcinolone injections and 2 intravitreal triamcinolone injections within 14 months with no complications. The CMO initially responded to each triamcinolone injection but later recurred (Figure 1A). 

The patient then received 4 intravitreal dexamethasone 0.7-mg implants (Ozurdex; Allergan, Inc.) uneventfully within 15 months, which maintained a dry fovea for a longer period but the CMO recurred again (Figure 1B, C, D, E). She also received a trial of anti-vascular endothelial growth factor (Avastin) but there was no response. At that point, the patient decided that she no longer wanted repeated injections and decided to wait until her fund application to receive ILUVIEN implant as special case was approved.

**Figure 2:**
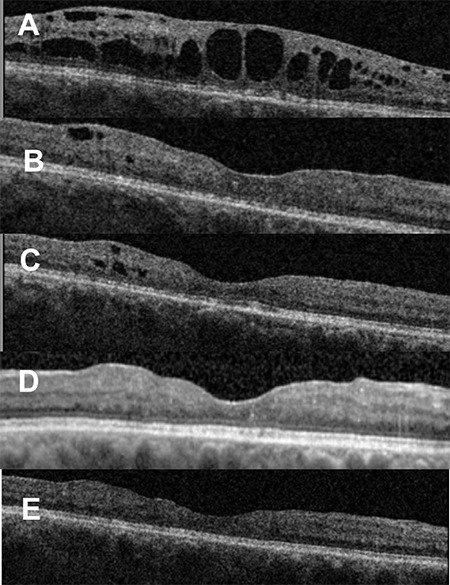
Optical coherence tomography images. A) Five months before ILUVIEN implant. B, C, D, E) Images taken 1, 6, 13, 20 months after ILUVIEN implant, respectively

Her refractory CMO persisted after 2 years without treatment. Finally, she received ILUVIEN intravitreal implant. In the first week she developed mild right eye anterior uveitis; IOP was 27 mmHg in the right eye and 18 mmHg in the left eye. These markedly regressed within a week on dexamethasone drops and latanoprost drops and topical medications were stopped within 4 weeks. At the time of this report, it is 20 months since receiving the ILUVIEN implant and she still has a dry fovea with right eye VA of 6/18 (Figure 2).

## Discussion

Refractory CMO after RD repair is difficult to treat and in a quarter of cases it will not improve without treatment. Intravitreal corticosteroid injections have shown to be an effective treatment option. We are not aware of any published literature in which ILUVIEN was used to treat this condition. This approach not only maintained an anatomical dry fovea but also provided visual improvement with a single ILUVIEN implant. This also demonstrates that ILUVIEN is an effective management strategy to reduce the need for repeated treatments. There is a risk of IOP elevation after receiving ILUVIEN intravitreal implant but in our case IOP was well controlled with short-term treatment. Further investigation of more cases with longer follow-up is needed. Better understanding of the exact aetiology of CMO after RD repair will lead to the development of more targeted treatment options.

## Figures and Tables

**Table 1:**
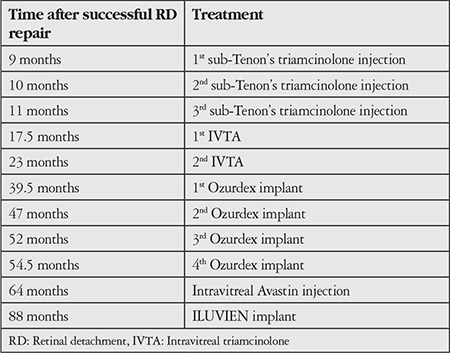
Summary of treatments for cystoid macular oedema during the follow-up period
